# CRISPR antiviral inhibits neurotrophic JC polyomavirus in 2D and 3D culture models through dual-gRNA excision by SaCas9

**DOI:** 10.1016/j.omtn.2025.102556

**Published:** 2025-05-14

**Authors:** Angela Rocchi, Shuren Liao, Hong Liu, Chen Chen, Senem Çakır, Anna Bellizzi, Hassen S. Wollebo, Ilker K. Sariyer, Kamel Khalili

**Affiliations:** 1 Department of Microbiology, Immunology and Inflammation, Center for Neurovirology and Gene Editing, Temple University Lewis Katz School of Medicine, Philadelphia, PA 19140, USA

**Keywords:** MT: RNA/DNA Editing, CRISPR, antiviral, gene editing, JC virus, progressive multifocal leukoencephalopathy, cerebral organoids

## Abstract

Without an effective antiviral, JC virus (JCV) has persisted throughout multiple epochs of immunosuppression, causing the opportunistic demyelinating disease, progressive multifocal leukoencephalopathy (PML). This study proposes a novel therapy using a dual-gRNA, SaCas9, CRISPR antiviral targeting JCV transcription factor, large tumor antigen (LT-Ag), and capsid protein, viral protein 1 (VP1). This treatment was validated using traditional two-dimensional cell culture. A recombinant cell line was produced from SVG astrocytes (SVGA) via lentiviral inoculation and puromycin selection. Following infection, sanger sequencing identified uniform excision of the circular dsDNA genome of JCV, significantly reducing viral load per genomic copy number on qPCR, viral proteins on western blot, and infectivity of viral progeny on adoptive transfer. Following this proof-of-concept using cell lines, translatability of results was advanced using three-dimensional, heterogeneous cerebral organoids (COs). COs were infected and treated with the lentivirus-packaged CRISPR antiviral. As observed in monolayer culture, a truncated genome was confirmed with sequencing, reducing viral load per genomic copy number on qPCR, protein levels on immunofluorescent imaging, and infectivity on adoptive transfer. The high efficacy of this JCV-targeting CRISPR antiviral in the context of cerebral organoids expounds on its value for the currently untreatable JCV and PML.

## Graphical abstract

**Figure:**
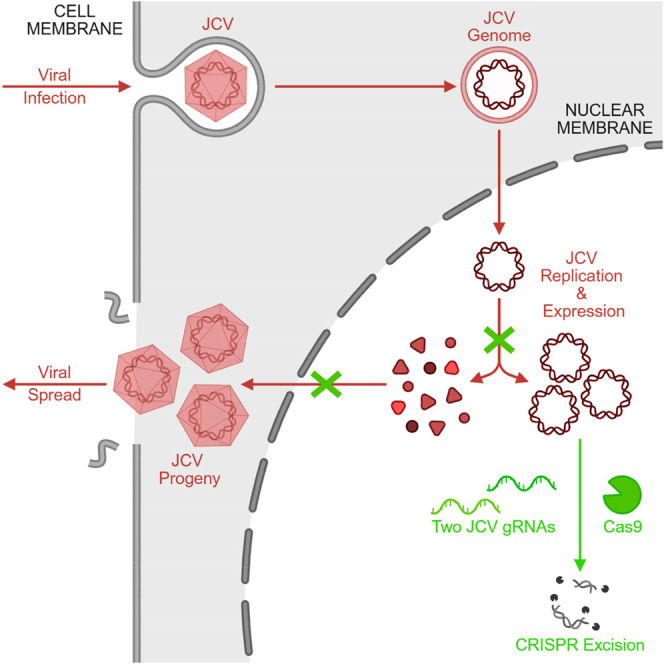

## Teaser

Khalili and colleagues develop a novel CRISPR antiviral to simultaneously target early and late genes of JC polyomavirus, excising the viral genome and suppressing pathogenic protein expression. Translatability of the evidence is enhanced with human cerebral organoids, a necessary advancement for the investigation of the currently untreatable JC polyomavirus.

## Introduction

Progressive multifocal leukoencephalopathy (PML) is an opportunistic disease wherein pathologic strains of JC virus (JCV) lyse oligodendrocytes, causing fatal demyelination of the central nervous system.^1^ Although the disease itself is rare (1 in 500,000 people annually), the underlying virus is endemic.^2^ Studies have found 50%–70% of healthy individuals are seropositive for anti-JCV antibodies, with 15%–20% shedding active JCV in urine.^3^^,^^4^ Although the cause of PML is well established, few advancements have been made in its treatment.^5^^,^^6^ Persistence of the opportunistic disease across multiple epochs of immunosuppression can be attributed to the standard of care: treatment of the immunosuppression rather than the virus itself.^7^^,^^8^^,^^9^^,^^10^ Here, we present the immense efficacy of a dual-gRNA, SaCas9, lentivirus-packaged CRISPR antiviral targeting the large tumor antigen (LT-Ag) and viral capsid protein 1 (VP1) open reading frames of the circular, dsDNA genome of JCV.

Utilization of gene editing to target viruses is a growing field of antivirals; CRISPR enables highly specific targeting of the DNA or RNA genomes of viruses.^11^^,^^12^ On delivery to a host—through viral capsid or nanoparticle packaging—the gRNA(s) and caspase are expressed by host polymerases. The gRNA(s) complement the target sequence and recruit the caspase to cleave upstream of a protospacer adjacent motif. At the time of writing, six clinical trials are ongoing for CRISPR targeting of human papillomavirus, human immunodeficiency virus, herpes simplex virus type I, Epstein-Barr virus, and coronavirus SARS-CoV-2 (clinicaltrials.gov). Pre-clinical investigations are more plentiful, targeting a range of viruses including influenza A, hepatitis B and C, dengue, and multiple orthopoxviruses.^13^^,^^14^^,^^15^^,^^16^^,^^17^

There are seven potential target regions of the circular dsDNA JCV genome: a noncoding control region (NCCR), small tumor antigen (smt-Ag), LT-Ag, agnoprotein (Agno), and viral proteins 1–3 (VP1–3).^18^ The NCCR is the bicistronic promoter and origin of the viral genome that produces two transcripts, early and late.^19^ The early transcript encodes small and large tumor antigen, which share an N-terminus via alternative mRNA splicing.^20^ LT-Ag uniquely exhibits helicase activity, a critical function that enables host polymerases to access the supercoiled circular dsDNA of JCV.^21^ Both smt-Ag and LT-Ag promote viral replication and expression, exhibiting myriad additional interactions with host cell proteins to facilitate the production of viral progeny. The later transcript produces Agno, a small viroporin, and three viral capsid proteins with overlapping reading frames.^22^^,^^23^^,^^24^ The major capsid protein, VP1, forms 72 pentamers, each surrounding a VP2 or VP3 monomer to comprise the non-enveloped icosahedral capsid.^25^^,^^26^

Multiple novel therapies for JCV have been proposed in the past; however, the absence of a translational model acts as a major barrier to research.^27^^,^^28^^,^^29^^,^^30^^,^^31^ Few cell types are permissive to JCV *in vitro*; cell culture modeling predominantly relies on the use of fetal astrocytes that have been transformed by the LT-Ag of analogous polyomavirus, simian virus 40.^32^ This cell line, SVGA, is permissive to the highly cell-specific JCV but also exhibits abnormal cell homeostasis that poorly recapitulates the normal human brain.^33^^,^^34^^,^^35^ Similarly permissive cells transformed by SV40 LT-Ag include simian kidney cells (COS-7) and human kidney cells (HEK), but these cells exhibit the same limitations of immortalization while deviating from the cells of interest in the CNS.^36^^,^^37^ Similarly, cells of neuroblast, choroid, and meningeal origin are infectible, but evidence suggests these tissues function as reservoirs and transition sites, rather than direct targets of PML pathogenesis.^38^^,^^39^^,^^40^^,^^41^ Using untransformed astrocytes or oligodendrocytes, the primary cells of interest in PML, is complicated by barriers in both infectability and legislation.^42^^,^^43^ Induced human cerebral organoids (COs) may offer a solution to these challenges.

Through stepwise stimulation of induced pluripotent stem cells (iPSCs), a multicellular three-dimensional organoid co-differentiates neurons and glia.^44^^,^^45^ The artificially produced cerebrum exhibits anatomical connections that mirror those of a natural brain including synaptogenesis and myelination.^46^^,^^47^^,^^48^ In 2022, Barreras et al. successfully infected COs with JCV via exposure to cerebral spinal fluid of patients with PML.^49^ Confocal imaging and electron microscopy confirmed the production of viral particles in CO glia. Given that COs originate in induced (not fetal) stem cells are not transformed by tumorigenic proteins and exhibit a multicellular anatomical environment, their susceptibility to JCV infection offers a major advancement in PML modeling. As such, this CO-infection model is the most translatable *in vitro* model for investigating JCV-targeting therapeutics to date. We employ this model to legitimize the preclinical value of our CRISPR antiviral, amplifying the significance of our results beyond proof-of-concept studies discussed in transformed cell lines.

Using *Benchling* bioinformatic software, we designed three gRNAs and compared the efficacy of seven potential combinations with SaCas9. Treatment with the dual-gRNA construct targeting LT-Ag and VP1 significantly reduced viral load. Stable cell lines expressing this construct were produced from the SVGA cell line using puromycin selection. Complete excision of the viral genome spanning the LT-Ag and VP1 sites was observed on excision assay. This reduced viral load in cell lysate and media, with reduced infectivity of viral progeny on adoptive transfer. Following validation with SVGA cells, results were replicated in a more translatable infection-treatment CO model. Although a majority of the target sequences were successfully excised, a minority population of full-length virus exhibited insertion or deletion mutations at one of the two gRNA sites. In summary, our results indicate CRISPR gene editing is a powerful platform to impair JCV infection in two- and three-dimensional cell culture models.

## Results

### Optimal CRISPR construct for reducing JC viral load concurrently targets LT and VP1 genes

**Figure 1:**
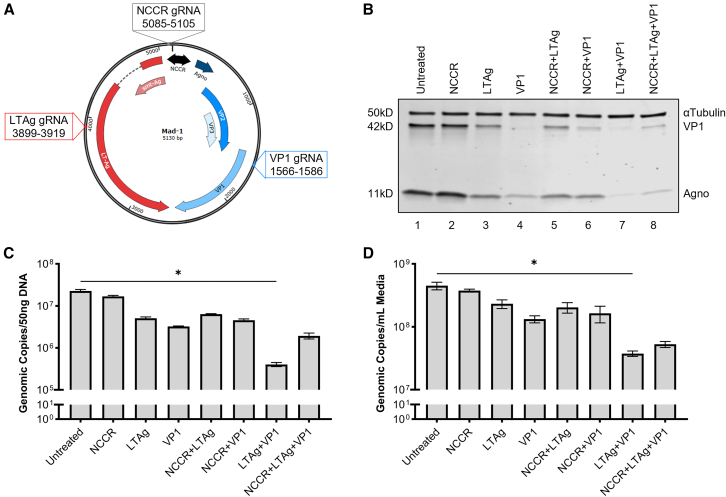
Dual-gRNA SaCas9 construct targeting JCV LT-Ag and VP1 produces the greatest reduction in JCV viral load (A) Map depicting optimal SaCas9-compatible gRNA targets on circular dsDNA genome of Mad-1 per bioinformatic software *Benchling*. Targeted regions include the NCCR (black), early transcript LT-Ag (red), and late transcript VP1 (blue). Seven separate combinations of these gRNAs were incorporated into SaCas9-construct, pPapi, to determine the most effective antiviral. (B) Western blot depicting viral protein levels of cells treated with CRISPR constructs described above. Compared to untreated control, the largest reduction in both VP1 (42 kD) and Agno (11 kD) is evident for cells treated with the two-gRNA construct targeting LT-Ag and VP1 (LTAg+VP1). (C) Viral genomic copy number measured by qPCR of DNA extracted from cell lysates of conditions described above reveals a statistically significant decrease in cells expressing the LTAg+VP1 dual-gRNA construct compared to untreated control (∗ = *p* = 0.0229, *n* = 16). (D) Viral genomic copy number measured by qPCR of DNA extracted from media of cell conditions described above reveals a statistically significant decrease in the media of the LTAg+VP1 dual-gRNA cells compared to untreated control (∗*p* = 0.0318, *n* = 16).

On review of the literature, three regions of the PML-causing strain of JCV (Mad-1) were identified as key to pathogenesis: the NCCR, early transcription factor LT-Ag, and primary capsid protein, VP1 (Figure 1A). The NCCR is responsible for the species and site specificity of the human polyomavirus, pathogenic variability of JCV strains, the propagation of viral progeny, and the expression of viral proteins.^50^^,^^51^ The positive-feedback mechanism of LT-Ag is an essential early regulator of viral gene replication and expression.^21^^,^^52^ In the absence of LT-Ag, JCV has been shown to exhibit significantly reduced viral load, signifying its critical role in viral propagation.^53^ The late transcript encodes VP1, a peptide that forms the 72-pentamer external capsid.^54^ In addition to protecting the viral genome during extracellular transport, VP1 interacts with 5HT2A receptors and sialic acid residues on host cells to facilitate endocytosis.^55^^,^^56^ Without VP1, viral progeny cannot be produced, nor can traditional viral infection be initiated at the cell membrane.^57^

Using the software *Benchling*, we searched the forementioned reading frames of the Mad-1 strain of the JCV genome published in the NCBI GenBank database (reference NC_001699.1) for DNA segments compatible with the SaCas9 PAM 5′-NNGRRT-3′. Off-target estimates were made in reference to cataloged human genome hg38. The gRNAs with the highest combined on-target efficacy and off-target safety scores were selected (Table S1). On-target sensitivity was calculated per activity scoring published by Doench et al.; off-target specificity was calculated per specificity scoring published by Hsu et al.^58^^,^^59^

Seven potential combinations of the three gRNAs were generated: three single-gRNA constructs, three dual-gRNA constructs, and one triple-gRNA construct. Using the lentiviral host plasmid pPapi originated by Najm et al., gRNAs were incorporated between a U6 promoter and a SaCas9-compatible gRNA scaffold.^60^ Each construct was packaged into a lentiviral vector for treatment of SVGA cells at a multiplicity of infection of 10^3^ viral particles per cell. Three days after transduction with the virus, cells were infected with the Mad-1 strain at a multiplicity of infection (MOI) of 10^2^ genomic copies per cell. At 14 days post-transduction and 11 days post-infection, cells and corresponding media were harvested for protein and DNA extraction. Viral proteins were observed by western blot using αTubulin as an intrinsic loading control (Figure 1B). Cells treated with the single gRNA construct targeting VP1 and the dual-gRNA construct targeting LT-Ag and VP1 exhibited visibly reduced VP1 bands, a viral gene directly targeted by both constructs. Only the dual construct targeting LT-Ag and VP1 successfully reduced the agnoprotein band as well. Agno is encoded upstream of the VP1 cleavage site (Figure 1A); this suggests the LTAg+VP1 construct successfully incapacitates expression of the entire viral genome, rather than cleavage sites alone. LT-Ag was not examined on western blot due to confounding by the high levels of SV40 LT-Ag that are endogenous to the SVGA cell line. Using qPCR, genomic copy number of cell lysate and media was compared across groups (Figures 1C and 1D). Compared to the untreated cells, a significant reduction in viral genomic copy number was observed in the DNA lysate and media of cells treated with the dual-gRNA construct targeting LT-Ag and VP1. This indicates that instability induced by the LTAg+VP1 construct occurs at the genomic level, upstream of the forementioned reduction in protein expression.

### Separation of gRNA cassettes improves expression of dual-gRNA SaCas9 construct

Given optimal bioinformatic analysis and an evident reduction in viral load, the dual-gRNA construct targeting JCV LT-Ag and VP1 was selected for further investigation (Figure S1A). To characterize construct expression, SVGA cells were transfected with the treatment construct and then harvested after 48 h. RNA and protein were extracted from cell lysate. Using reverse transcription qPCR, gRNA levels were compared using primers for the LT-Ag and VP1 gRNA cassettes (Table S2). A significantly higher level of VP1 gRNA was observed relative to LT-Ag gRNA, suggesting an expression-dependent disparity in gRNA levels in a clonally expanded cell line (Figure S1C). Both gRNAs utilize the U6 promoter; therefore, transcription is performed by RNA polymerase III.^61^ Being directly in parallel, with the LT-Ag gRNA scaffold directly proceeding the VP1 gRNA U6 promoter, concerns of steric interference arose.^62^

To address this, a second treatment construct originating from the same Cas9-only parental plasmid, pPapi, was designed, with the LT-Ag gRNA preceding the SaCas9 reading frame and the VP1 gRNA succeeding it (Figure S1B). SVGA cells transfected with this “Split” construct were compared to cells transfected with the original construct where gRNAs were ordered in “Tandem.” A 3-fold increase in gRNA expression was observed for both LT-Ag and VP1 gRNAs (Figure S1C). A greater level of VP1 gRNA was still observed; however, this difference was not significant. No significant change in SaCas9 expression was observed at the RNA or protein level when transfected with the parent plasmid expressing SaCas9 without gRNAs (pPapi), the construct with LT-Ag and VP1 gRNAs in tandem, or the rearranged construct with LT-Ag and VP1 gRNAs split by the SaCas9 cassette (Figures S1D–S1F).

### Cells inoculated with dual-gRNA treatment construct targeting JCV LT-Ag and VP1 excise linking genome, interrupting viral replication

In the pPapi plasmid, the SaCas9 reading frame is shared with a puromycin resistance gene under the elongation factor 1α promoter (Figure S1A). We utilized this feature to transduce SVGA cells with either the parental pPapi (SVGA+Cas9) or the split dual-gRNA construct targeting JCV LT-Ag and VP1 (SVGA+Cas9+gRNAs). Cells were then maintained in media enriched with puromycin (1 μg/mL) such that only cells expressing the construct were expanded. After expansion, a subpopulation of cells was harvested for RNA and protein extraction. Quantification of gRNAs and SaCas9 mRNA confirmed construct expression on RT-qPCR (Figures S2A and S2B). Expression of Cas9 protein was confirmed and quantified by western blot (Figures S2C and S2D).

**Figure 2:**
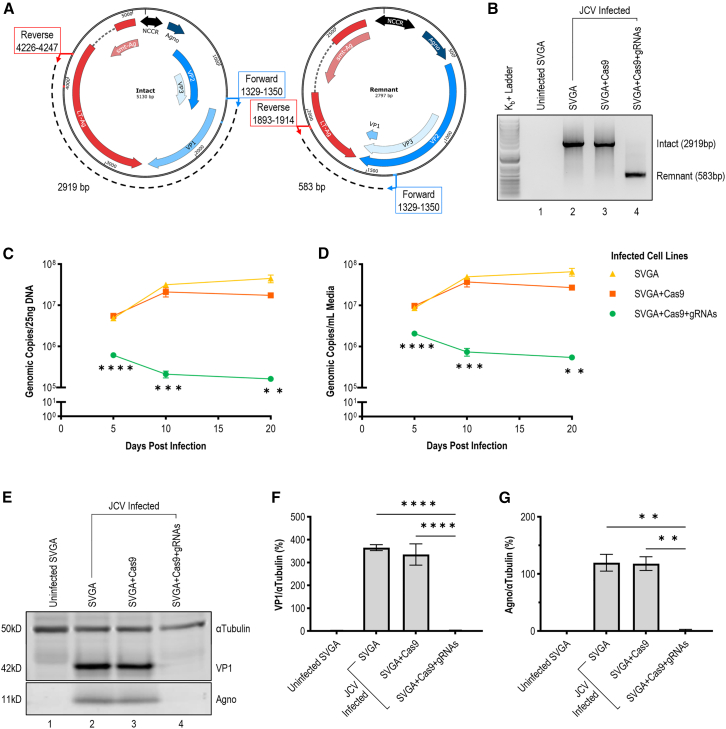
Excision of JCV genome linking LT-Ag and VP1 cut sites by dual-gRNA CRISPR construct reduces viral genomic copy number of cells and media and eliminates viral protein expression (A) Illustration of ePCR amplicons produced by primers bound to full-length (“Intact”) and CRISPR-excised (“Remnant”) Mad-1. A full-length genome produces a 2,919 bp amplicon; following excision of the region linking gRNA cut sites, a 583 bp amplicon (green) is produced. (B) Gel electrophoresis of ePCR described above using DNA from cell lysate of uninfected parental cell line (1), infected parental cell line (2), infected SVGA cells selected for Cas9-only construct expression (3), or infected SVGA cells selected for full construct expression with Cas9 and LTAg+VP1 gRNAs (4). Harvesting was performed 5 dpi. A full-length intact genome is evident for infected parental and Cas9-only lines; no residual intact band is present for the treatment-expressing cell line. Sanger sequencing confirmed bands align with the predicted amplicons discussed above. (C) Time course qPCR of viral DNA extracted from cell lysate of conditions described above at 5, 10, and 20 dpi with Mad-1. Compared to infected parental (▲) and Cas9-only (■) lines, a significant decrease in *intracellular* viral load was observed at day 5 (∗∗∗∗*p* < 0.0001, *n* = 16), day 10 (∗∗∗*p* = 0.0002, *n* = 16), and day 20 (∗∗*p* = 0.0067, *n* = 16) in the treatment-expressing (●) cell line. (D) Time course qPCR of viral DNA extracted from media accompanying cell lines described above. Compared to infected parental (▲) and Cas9-only (■) lines, a significant decrease in *extracellular* viral load was observed at day 5 (∗∗∗∗*p* < 0.0001, *n* = 16), day 10 (∗∗∗*p* = 0.0001, *n* = 16), and day 20 (∗∗*p* = 0.0052, *n* = 16) compared to infection controls. (E) Western blot displaying viral proteins VP1 (42 kD) and Agno (11 kD) relative to αTubulin (50 kD) loading control at endpoint harvest (20 dpi) of cell lysates described above. (F) Quantification of western blot described above shows significant decrease in VP1 of treatment-expressing cells when compared to Cas9-only control (∗∗∗∗*p* < 0.0001, *n* = 16). No significant difference between infected treatment-expressing cells and never-infected control (*p* > 0.9999, *n* = 16). (G) Quantification of western blot described above shows significant decrease in Agno of treatment-expressing cells when compared to Cas9-only control (∗∗*p* = 0.0090, *n* = 16). No significant difference between infected treatment-expressing cells and never-infected control (*p* > 0.9999, *n* = 16).

Cells of the forementioned lines were infected with Mad-1 at MOI = 5 genomic copies per cell and maintained in normal media for 20 days. To determine if the dual-gRNA construct successfully excises the linking region of the circular viral genome, DNA extracted from cell lysate 5 days post-infection (dpi) was subjected to excision PCR (ePCR) using complementary primers upstream of the LT-Ag and VP1 gRNA target sites (Table S2). This ePCR effectively produces a 2919 base pair (bp) amplicon when full-length Mad-1 is “intact” (Figure 2A, left). Alternatively, concurrent cleavage of both sites enables removal of the linking region, with recircularization of the cleaved ends producing a visibly shorter “remnant” amplicon of 583 bp (Figure 2A, right). On gel electrophoresis, a distinct excised, remnant band was observed with no full-length, intact band (Figure 2B). Both intact and remnant ePCR amplicons were confirmed by Sanger sequencing. No residual intact band was identified for the treatment-expressing cell line, suggesting complete inoculation against Mad-1 by the LTAg+VP1 dual-gRNA construct.

DNA extracted from cell lysate at days 5, 10, and 20 post-infection indicated a significant reduction in viral load of treatment-expressing cells compared to infected Cas9-only and parental SVGA control lines (Figure 2C). DNA extracted from cell media at these timepoints showed a similar reduction (Figure 2D). Furthermore, while infected control lines exhibited an upward trend and plateau in viral load, the treatment-expressing line exhibited a downward trend. Simple linear regression confirmed an upward trend of both infection controls over time (R^2^ = 0.6357); with an opposing downward trend observed for the treatment-expressing cell line (R^2^ = 0.6295). Comparison of slopes was performed via a two-tailed t test, which determined the difference generated was extremely significant (*p* = 0.0004). This indicates the rate of degradation of the viral genome by the preexisting treatment construct exceeded the rate of viral replication of the Mad-1 strain.

The reduction in viral load was most evident at the protein level, with no VP1 or agnoprotein visible on western blot compared to infected control groups (Figure 2E). Compared to an αTubulin loading control, the treatment group was not significantly different compared to uninfected SVGA cells, suggesting complete suppression of viral protein expression within the metrology of WB (Figures 2F and 2G). Given the truncation of the viral genome, we sought to further illustrate the ability of the treatment construct to terminate, rather than suppress, the viral life cycle of JCV.

**Figure 3:**
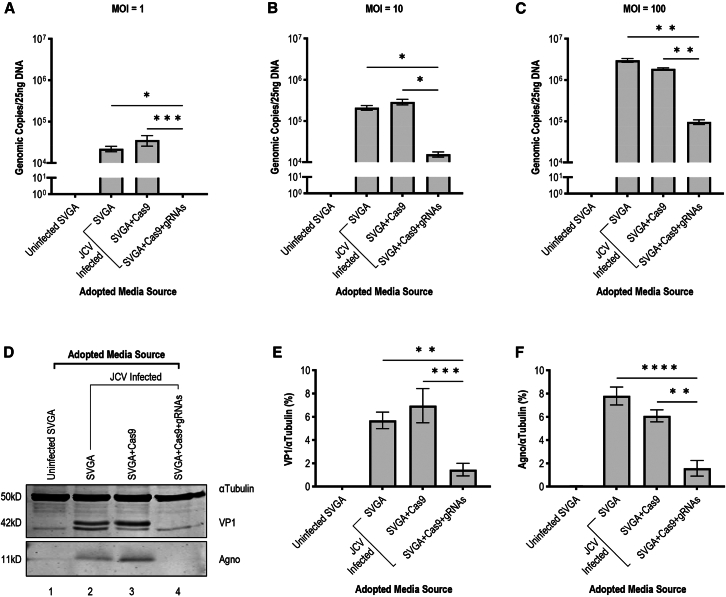
Adoptive transfer of infected cell media demonstrates impaired infectivity of viral progeny produced by treatment-expressing cells (A) Adoptive transfer of media from uninfected parental, infected parental, infected Cas9-only, and infected treatment-expressing cell lines to treatment and infection naive SVGA cells at MOI = 1 genomic copy per cell. Following 10 days of incubation, media transferred from treatment-expressing cell lines resulted significantly fewer JCV genomic copies on qPCR compared to those media transferred from parental (∗*p* = 0.0144, *n* = 15) and Cas9-only expressing (∗∗∗∗*p* = 0.0007, *n* = 15) lines. Conversely, no significant change was observed when compared to adoptive transfer from uninfected controls (*p* > 0.9999, *n* = 15). (B) Adoptive transfer described above was replicated at MOI = 10. A significant difference is observed in the viral load of SVGA cells following adoptive transfer from treatment-expressing cells compared to adoptive transfer from parental (∗*p* = 0.0216, *n* = 16) and Cas9-only expressing (∗*p* = 0.0318, *n* = 16) lines. (C) Adoptive transfer described above was replicated at MOI = 100. A significant difference is observed in the viral load of SVGA cells following adoptive transfer from treatment-expressing cells compared to adoptive transfer from parental (∗∗*p* = 0.0099, *n* = 16) and Cas9-only expressing (∗∗*p* = 0.0013, *n* = 16) lines. (D) Western blot displaying viral proteins VP1 (42 kD) and Agno (11 kD) relative to αTubulin (50 kD) loading control of cell lysate of conditions described above following adoptive transfer at MOI = 100. (E) Quantification of WB described above reveals a significant decrease in VP1 following adoptive transfer of media from treatment-expressing cells compared to adoptive transfer from infected parental (∗∗*p* = 0.0022, *n* = 16) and Cas9-only (∗∗∗*p* = 0.0001, *n* = 16) cells. Compared to adoptive transfer from uninfected controls, transfer from treatment-expressing cells produced no significant difference (*p* = 0.4492, *n* = 16). (F) Quantification of WB described above reveals a significant decrease in Agno following adoptive transfer of media from treatment-expressing cells compared to adoptive transfer from infected parental (∗∗∗∗*p* < 0.0001, *n* = 16) and Cas9-only (∗∗*p* = 0.0011, *n* = 16) cells. Compared to adoptive transfer from uninfected controls, transfer from treatment-expressing cells produced no significant difference (*p* = 0.4527, *n* = 16).

Using media harvested from the previous experiment at 20 dpi, parental SVGA cells were exposed to media from the four groups described above. Three MOIs (1, 10, and 100 genomic copies per cell) were applied using concentrations measured for each condition via qPCR. Cells were harvested 10 days after media transfer. DNA and protein were extracted from cell lysate. Viral load by qPCR of genomic copy number was significantly reduced at all three MOIs for cells treated with media produced by gRNA-expressing cell lines compared to those treated with media from infected parental and Cas9-only lines. At MOI = 1, no viral copies were measured in treatment-naïve cells after adoptive transfer of the treated cell line, making it indistinguishable from the uninfected control (Figure 3A). At MOI = 10 and MOI = 100, a 16-fold and 25-fold reduction were observed, respectively (Figures 3B and 3C). At the protein level, this reduction was even more drastic with no viral proteins observed on western blot at the highest MOI (Figures 3D–3F). This suggests the viral genome editing of JCV LT-Ag and VP1 not only reduces the viral expression and reproduction in directly treated cells but also reduces the infectability of viral progeny, reducing the risk of infection for untreated bystander cells. By producing a significant decrease in viral load of untreated cells through adoptive transfer, we illustrate the high efficacy of the treatment construct targeting JCV LT-Ag and VP1 at interrupting the viral life cycle, in addition to preventing its expression-linked pathogenesis.

### Characterization of COs infected with Mad-1 provides a more translatable model of JCV

The development of COs from human iPSCs is well established.^46^ After 40 days, mature organoids consistently exhibit astrocytes and neurons.^63^ After approximately 80 days, oligodendrocytes are observable.^47^ We produced COs through stepwise exposure of iPSCs to developmental transcription factors, exogenous synthetic basal membrane, and centripetal force such that codifferentiation of neurons and glia occurs in a three-dimensional spheroid (Figure S3A). We processed 120-day-old COs to confirm the presence of neurons, astrocytes, and oligodendrocytes (Figures S3D–S3D). Electron microscopy obtained of COs of the same age identified spiraling membranes characteristic of myelin at different stages of packing (Figure S3E).

**Figure 4:**
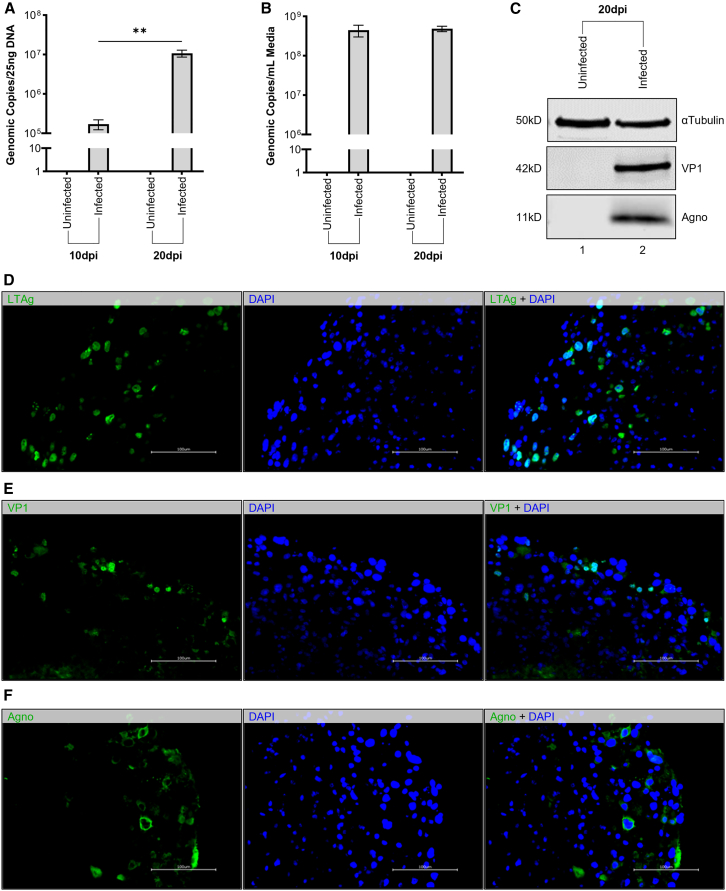
COs successfully replicate viral DNA and express viral proteins following infection with Mad-1 strain of JCV (A) JCV genomic copy number on qPCR of CO lysate DNA following infection with Mad-1 at MOI = 10. Significant difference observed between infected COs across time (∗∗*p* = 0.0025, *n* = 12). Average viral load was 1.719 × 10^5^ genomic copies 10 dpi, which increased to 1.079 × 10^7^ genomic copies 20 dpi, suggesting successful propagation of JCV progeny by host COs. (B) JCV genomic copies per mL media of COs described above via qPCR. Average viral load of 4.470 × 10^8^ genomic copies 10 dpi only slightly increased to 4.867 × 10^8^ genomic copies 20 dpi (*p* = 0.6474, *n* = 24), suggesting extracellular concentration of viral DNA does not directly correlate with intracellular viral load of healthy, heterogeneous cell populations of COs. (C) Western blot showing distinct bands positive for viral proteins VP1 (42 kD) and Agno (11 kD) in COs 20 dpi with Mad-1 compared to uninfected control despite equivocal αTubulin (50 kD) loading control. (D) Immunohistochemistry of COs described above displaying strong immunofluorescent tagging of viral transcription factor LT-Ag (green) uniformly overlapping with DAPI nuclear stain (blue). (E) Immunohistochemistry of COs described above displaying faint immunofluorescent labeling of viral capsid VP1 (green) overlapping with DAPI nuclear stain (blue) predominantly in the peripheral cell mass with poor penetrance relative to LT-Ag discussed above. (F) Immunohistochemistry of COs described above presents strong immunofluorescence for disseminated perinuclear viral protein Agno (green) surrounding more discrete DAPI nuclear stain (blue). Known to be released and absorbed by adjacent cells, the more diffuse signal illustrates the protein’s ability to identify both actively infected and passive bystander cells.

The susceptibility of mature COs to host the Mad-4 strain of JCV has been reported.^49^ To generate an infection model, COs were exposed to Mad-1 at MOI = 10 genomic copies per cell, estimating 1 × 10^6^ cells per organoid based on previous cell counting of a subpopulation of COs at this age.^63^ Viral DNA was quantifiable in the lysate and media of COs at 10 dpi (Figure 4A). At day 20, a significant increase in viral genomic copy number of lysate DNA was observed on qPCR, whereas a minor increase was observed for corresponding media (Figure 4B). We posit that this is a result of the continuous centrifugation required for CO maintenance: in redirecting released viral progeny back toward the cell mass, reinfection may trap virions that would be observed in static monolayer culture. Furthermore, the spheroid structure of COs allows virions to directly infect adjacent cells in three dimensions, rather than infecting in two dimensions wherein the third dimension is occupied by media. Western blot of protein lysate harvested at 20 dpi produced strong bands for both VP1 and Agno compared to an uninfected control population from the same CO differentiation sect (Figure 4C). LT-Ag, VP1, and Agno were observable via immunofluorescence with halo-like infiltration of the outer cell mass (Figures 4D and 4E).

**Figure 5:**
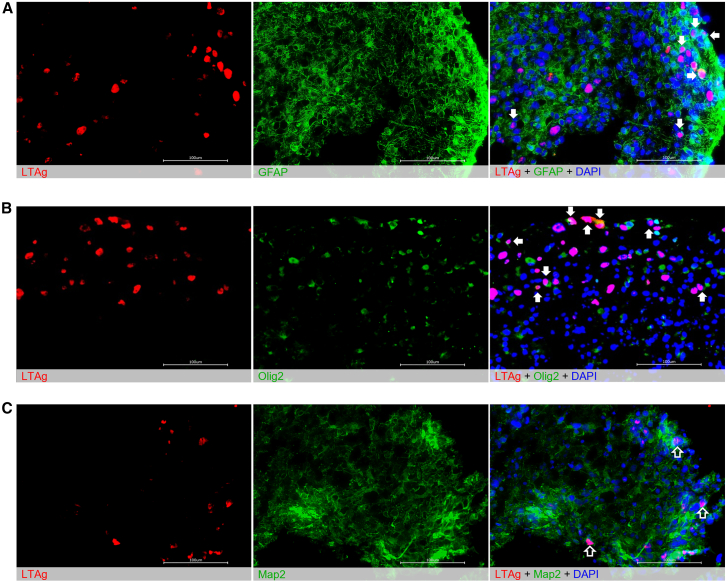
Mad-1 strain of JCV preferentially infects astrocytes and oligodendrocytes in CO model (A) Immunohistochemistry of COs 20 dpi with Mad-1 strain of JCV displaying strong immunofluorescent tagging of active viral infection (LT-Ag, red) and astrocyte processes (GFAP, green). Overlapping regions (white arrows) indicate infected astrocytes. Nonspecific nuclear staining (DAPI) supports the presence of a heterogeneous cell population. (B) Immunohistochemistry of COs 20 dpi with Mad-1 strain of JCV displaying strong immunofluorescent tagging of active viral infection (LT-Ag, red) and oligodendrocyte nuclei (Olig2, green). Overlapping regions (white arrows) indicate infected oligodendrocytes. Nonspecific nuclear staining (DAPI) supports the presence of a heterogeneous cell population. (C) Immunohistochemistry of COs 20 dpi with Mad-1 strain of JCV displaying strong immunofluorescent tagging of active viral infection (LT-Ag, red) and neuron processes (Map2, green). The absence of strong overlapping regions (empty arrows) suggest poor infectivity of neurons. Nonspecific nuclear staining (DAPI) supports the presence of a heterogeneous cell population.

To further elucidate the translatability of the heterogeneous CO model, co-staining was performed using cell-type-specific markers and viral transcription factor LT-Ag. Compared to a general nuclear stain (DAPI), robust infection of astrocytes (Figure 5A) and oligodendrocytes (Figure 5B) was observed 20 dpi. Conversely, sparing of neurons was observed (Figure 5C). This is consistent with both basic science and clinical studies, which have previously shown glial preference for a majority of JCV strains, with specific VP1 mutations required to induce neuronopathy.^64^^,^^65^^,^^66^^,^^67^^,^^68^ This supports the use of COs as a more translatable *in vitro* model for JCV infections of the brain.

### Viral load is significantly reduced in Mad-1-infected COs following treatment with lentivirus-packaged, dual-gRNA CRISPR construct targeting JCV genes LT-Ag and VP1

**Figure 6:**
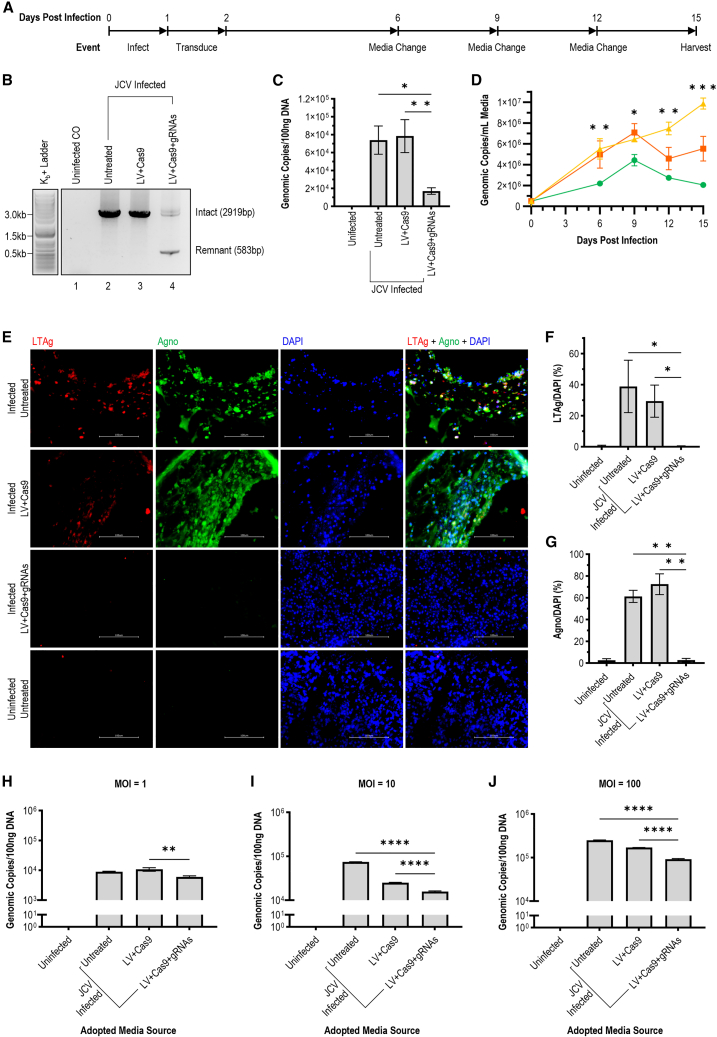
Infected COs exhibit significantly reduced viral load when treated with lentivirus-packaged CRISPR construct targeting JCV LT-Ag and VP1 (A) Time course of experimental design depicts infection (day 0), treatment (day 1), media harvests (days 6, 9, 12, and 15), and endpoint harvesting of lysates and whole CO fixation (day 15). (B) Gel electrophoresis of ePCR for DNA lysate from uninfected, untreated COs (1); infected, untreated COs (2); infected COs transduced with Cas9-only lentivirus (3); infected COs transduced with the dual-gRNA CRISPR lentivirus (4). A 2919 bp amplicon indicates full-length bands for infected controls. A full-length band and a 583 bp remnant band are visible for the treatment group, indicating majority excision. (C) Viral genomic copy number observed by qPCR of DNA lysate of conditions described above. Treated COs exhibited a significant decrease compared to infected, untreated COs (∗*p* = 0.0176, *n* = 24) and infected, Cas9-only treated COs (∗∗*p* = 0.0100, *n* = 24). (D) Viral genomic copy number observed by qPCR of DNA extracted from media of conditions described above. Compared to infection controls, treated COs exhibited a significant decrease at each of measured timepoints: day 6 (∗∗*p* = 0.0066, *n* = 21), day 9 (∗*p* = 0.0166, *n* = 21), day 12 (∗∗*p* = 0.0020, *n* = 21), and day 15 (∗∗∗*p* = 0.0001, *n* = 21). (E) Immunofluorescent imaging of CO conditions described above displaying viral nuclear transcription factor LT-Ag (red) and perinuclear Agno (green) relative to DAPI nuclear stain (blue). (F) Quantification of fluorescent images described above shows a significant reduction of LT-Ag in the treatment group when compared to untreated (∗*p* = 0.0182, *n* = 20) and Cas9-only (∗*p* = 0.0294, *n* = 20) COs, resulting in fluorescence comparable to the never-infected CO controls (*p* > 0.9999, *n* = 20). (G) Quantification of fluorescent images described above shows a significant reduction of Agno in the treatment group when compared to untreated (∗∗*p* = 0.0077, *n* = 22) and Cas9-only (∗∗*p* = 0.0038, *n* = 22) COs, resulting in fluorescence comparable to the never-infected CO controls (*p* > 0.9999, *n* = 22). (H) Viral load of DNA lysate following adoptive transfer of media from the above CO groups at MOI = 1 genomic copy per parental SVGA cell. A significant reduction was observed in infectivity of viral progeny produced by CRISPR treated COs compared to Cas9-only treated infection controls (∗∗*p* = 0.0037, *n* = 16). (I) Viral load of DNA lysate following adoptive transfer of media from the above CO groups at MOI = 10 genomic copies per parental SVGA cell. A significant reduction was observed in infectivity of viral progeny produced by CRISPR-treated COs compared to untreated (∗∗∗∗*p* < 0.0001, *n* = 16) and Cas9-only treated (∗∗∗∗*p* < 0.0001, *n* = 16) infection controls. (J) Viral load of DNA lysate following adoptive transfer of media from the above CO groups at MOI = 100 genomic copies per parental SVGA cell. A significant reduction was observed in infectivity of viral progeny produced by CRISPR-treated COs compared to untreated (∗∗∗∗*p* < 0.0001, *n* = 16) and Cas9-only treated (∗∗∗∗*p* < 0.0001, *n* = 16) infection controls.

To advance the translatability of our investigation, we employed an infection-treatment time course using COs with lentivirus-packaged CRISPR delivery (Figure 6A). In addition to the advanced three-dimensional, multicellular model human COs offer, a therapeutic intervention more appropriately models patient presentation as PML requires onset of clinical symptoms prior to diagnosis and treatment.^69^ A post-infection treatment model is, therefore, more translatable than prophylactic inoculation. COs matured 50 days post-differentiation from iPSCs were uninfected or infected with Mad-1 at MOI = 10 genomic copies per cell. Infected organoids were then untreated, transduced with Cas9-only control lentivirus (LV + Cas9), or transduced with the complete CRISPR lentivirus targeting LT-Ag and VP1 (LV + Cas9+gRNAs). COs were maintained for 15 days with regular media harvested during routine changes for time course analysis. RT-qPCR of endpoint RNA (harvested 15 dpi) confirmed gRNA and SaCas9 expression (Figure S4).

Using the previously designed ePCR excision assay (Figure 2A), DNA extracted from CO lysate 15 dpi displayed no band for the uninfected Cos; significant full-length bands for infected, untreated, and infected Cas9-only treated groups; and both intact and residual bands for the infected, full construct-treated COs (Figure 6B). Whereas the full-length band for untreated and Cas9-only conditions aligned with the expected intact Mad-1 sequence, and the shortened amplicon of the treatment condition aligned with the expected remnant band, the residual full-length band observed for the treatment group exhibited a mixed population. Additional primers were developed downstream of the LT-Ag and VP1 excision sites to capture target regions of non-excised amplicons (Table S2). Sanger sequencing was performed for use in Inference of CRISPR Edits (ICE) analysis with Synthego software. Of the un-excised viral genomes of the treated COs, 12% of the LT-Ag gRNA sites and 21% of the VP1 gRNA sites were mutated with multiple minority populations exhibiting insertions or deletions. This indicates that, even with the reduced concentration of the full-length ePCR amplicon, a percentage of the residual intact band may be non-functional.

DNA extracted from CO lysate at harvest day 15 showed a significant reduction in the treatment group, with an average decrease of 80% genomic copies per 100 ng DNA (Figure 6C). A significant difference was identified between infection controls (Untreated and LV + Cas9) and treated (LV + Cas9+gRNAs) COs, but no difference between treated COs and the uninfected control. Media DNA extracted at each media change was analyzed by qPCR. Infection controls produced significantly more virions at each time point when compared to the CRISPR-treated group (Figure 6D). As described with the inoculated cell lines, the treated COs exhibited a downward plateau in viral production, whereas the infected controls exhibit an upward trend insignificant from one another. The upward trend generated by infection controls (R^2^ = 0.6357) was eliminated in the treatment group (R^2^ = 0.0295) per simple linear regression, producing a statistically significant difference in trend per two-tailed t test (*p* = 0.0038). Considering the flawed correlation of intracellular viral load of COs to the respective extracellular media that was observed during the generation of the CO model (Figure 4B), this shift is remarkable although not as drastic as the inverted trend observed in the prophylactically treated cell line study (Figure 2D).

Whole organoids were sectioned for immunofluorescent labeling of LT-Ag and Agno in each condition. Both viral proteins were quantified and normalized using DAPI nuclear stain. At 15 dpi, both proteins were visible in infected COs that were untreated or treated with Cas9-only lentivirus (Figure 6E). This was significantly higher than the treatment group, which was largely indistinguishable from the uninfected CO group (Figures 6F and 6G). This stark contrast on immunofluorescent imaging mirrors the band differences seen on western blot in preceding cell line studies (Figures 2E–2G). This is logical in the context of the previous CO infection study (Figures 4 and 5), as COs are less capable of producing JC viral progeny when compared to the transformed SVGA line, wherein endogenous SV40 LT-Ag promotes proliferation by co-stimulating the JCV promoter.^32^^,^^33^^,^^34^^,^^35^^,^^36^

Media harvested from COs in each of the four conditions described was quantified 15 dpi for adoptive transfer to SVGA cells at MOI = 1, 10, or 100 genomic copies per cell. After 10 days of incubation, cell lysate was harvested for DNA analysis of viral load. Cells exposed to media adopted from infected COs that were treated with the CRISPR lentivirus targeting LT-Ag and VP1 exhibited significantly fewer genomic copies compared to those exposed to media adopted from infected, untreated, or Cas9-only-treated COs at all MOI (Figures 6H–6J). At MOI = 1, an average reduction of 33% was observed (Figure 6H), at MOI = 10 an average reduction of 80% was observed (Figure 6I), and at MOI = 100 an average reduction of 63% was observed (Figure 6J).

In addition to the reduction in infectivity seen in virions produced by COs treated with the dual-gRNA expressing lentivirus, a significant reduction was also evident for COs treated with the Cas9-only control lentivirus at MOI = 10 and MOI = 100. This suggests transduction with the lentivirus or the presence of SaCas9 is sufficient to impair viral progeny produced by non-transformed, heterogeneous human cerebral cells. We theorize that the mechanism by which this occurs is through manipulation of the protein quality control pathway, with processing of foreign proteins altering the host cell’s ability to process and release active virions. Proteosome inhibitors have previously been proposed as antiviral agents for multiple viruses, as their alteration of host proteostasis impairs viral replication.^70^^,^^71^^,^^72^ Studies have shown JCV manipulates both the proteasomal and lysosomal management of cell homeostasis via agnoprotein-dependent sequestration.^73^^,^^74^ Competing with agnoprotein, processing of exogenous SaCas9 may overwhelm the protein quality control mechanisms of a cell.^75^^,^^76^ The resultant build-up of intracellular virions (evidenced by increased intracellular viral load on Figure 6C and increased agnoprotein signal on Figures 6E and 6G) leads to the production of infectious virions into media (evidenced by lower viral load of media on Figure 6D). It is important to note that this impairment in the production of viral progeny is significantly less than the impairment observed following treatment with the full, gRNA-containing construct (Figures 6H–6J). The absence of an SaCas9-dependent effect in earlier SVGA lines can be explained by aberrant protein quality control mechanisms of transformed, immortalized cell lines.^33^^,^^34^^,^^35^ This re-emphasizes the importance of more translatable *in vitro* modeling for JCV.

## Discussion

The currently unmet need for a JCV antiviral is evidenced by recurrent surges in PML incidence during epochs of immunosuppression.^6^ Whereas genetic causes of immunosuppression (e.g., leukemia and agammaglobulinemia) remain steady, acquired causes vary based on fluctuating environmental pressures (e.g., HIV/AIDS).^77^ At present, increasing prescription of immune-targeting medications for the treatment of autoimmune conditions has increased the risk of iatrogenic PML.^78^ Autoimmune conditions affect 10% of the general population, with the annual rate of incidence continuing to rise.^79^ For these patients suffering serious conditions (like leukemias/lymphomas, multiple sclerosis, post-transplant rejection, and systemic lupus erythematous), immune suppression is both therapeutic and essential; associated medications (rituximab/cyclophosphamide, natalizumab, mycophenolate mofetil, and high-dose corticosteroids) increase PML risk by increasing the likelihood of acquiring JCV, enabling mutation of JCV into neurovirulent strains, and/or reactivating already-present JCV to lyse oligodendrocytes.^80^^,^^81^^,^^82^^,^^83^ JCV is quiescent in a majority of the population; various genetic and environmental pressures can induce its opportunistic shift toward PML.^1^^,^^2^^,^^3^^,^^4^^,^^5^ This makes the production of a highly effective JCV antiviral essential, as JCV is the sole, uniform cause of a diverse patient population.^84^^,^^85^^,^^86^^,^^87^

Here, we used two-dimensional and three-dimensional cell culture to prove that a dual-gRNA CRISPR antiviral effectively targets JCV LT-Ag and VP1 sites (Figure 1). The expression of SaCas9 and JCV-specific gRNAs by SVGA clonal cell lines showed the generation of target-specific deletions (2336 bp) of the viral genome (Figures 2A and 2B). Excision of the viral genome (Figure 2B), elimination of viral proteins (Figure 2C), and reduced infectivity of viral progeny (Figure 3) are unprecedented when compared to previous efforts to design a CRISPR antiviral, which show only a reduction in viral protein expression and single-site mutations from non-homologous end-joining.^28^^,^^29^ CO modeling of JCV is revolutionary in the field; although infection of COs with Mad-4 has been achieved in the past, use of this model to advance the translatability of a JCV therapeutic is novel.^49^ Using lentivirus as a delivery vector, heterogeneous COs were successfully treated after initiation of infection, supporting the value of the CRISPR antiviral as a potential *therapeutic* treatment, in addition to the *prophylactic* treatment observed in the stable cell line, proof-of-concept study (Figures 6 and 2, respectively). In the minority population of full-length genomic copies, a significant portion exhibited insertion and deletion mutations within the gRNA-targeted LT-Ag (12%) and VP1 (21%) open reading frames (Figure 6B). In addition to direct reduction of viral DNA and protein expression (Figures 6C–6G), reduced infectivity of viral progeny was observed by adoptive transfer (Figures 6H–6J). Use of the SV40 LT-Ag-primed SVGA line overestimates the proliferation capacity for JC viral progeny.^32^^,^^33^^,^^34^^,^^35^^,^^36^ The greater production of infectious virions by treated COs relative to inoculated cell lines could also be attributed to the minority population of functional intact virus (Figure 6B). Further optimization of treatment titer, two-phase rather than single-treatment timing, or alternate CRISPR delivery vectors could improve the treatment to ensure elimination of the viral genome and termination of the pathogenic life cycle.

Clinical development of this methodology will necessitate the use of advanced and sophisticated techniques for gene delivery. Promising peripheral delivery vectors include self-inactivating lentivirus and adeno-associated virus 9, which readily cross the blood-brain barrier.^88^^,^^89^^,^^90^^,^^91^ To avoid potential immune activation by the therapy, intraparenchymal delivery can also be considered for severe cases where brain biopsy or surgical manipulation is indicated.^92^ Our treatment plasmid is 12,482 bp (Figure 1B) and produces two 7,612 bp ssRNA bands that require the larger viral capsid lentivirus.^93^ The pPapi lentivirus was selected for its larger carrying capacity, its strong penetrance of the CNS, and the presence of the puromycin resistance gene.^60^ Additional benefits to using lentivirus, rather than full-size adenovirus, include a reduced immune response by the host and integration into both actively dividing (i.e., astrocytes) and non-dividing (e.g., oligodendrocytes) cell types.^94^^,^^95^ Associated risks of integration by the lentiviral construct do require thorough analysis of the host genome to determine any off-target genetic instability due to integration.^96^^,^^97^ This is a necessary investigation that warrants consideration in a multi-system context, as non-cerebral cell types exhibit alternative epigenomic conditions that could produce off-target effects not foreseen by the preliminary bioinformatic design.^98^ The high fidelity of SaCas9 minimizes the risk of off-target cleavage, but ultimate safety of the construct must consider collateral cleavage in multiple systems.^99^ Despite the advances provided by COs, the novelty of the CRISPR antiviral requires the extensive multi-system investigation a small animal model provides.^100^

Nonintegrating viruses (i.e., AAV9) can be considered, but the reduced risk may be accompanied by reduced therapeutic value.^101^ The patient population at risk for iatrogenic PML is growing, with treatments for cancers and autoimmune conditions relying on immunosuppression; use of integrating lentivirus allows introduction of the CRISPR antiviral prior to the onset of prescribed immunosuppression, preventing the irreversible damage caused by JCV progression to PML.^78^^,^^80^ Other preventative measures, such as vaccination, have been proposed, but are weakened by the poor immunogenicity of JCV as well as the opportunistic nature of PML.^102^^,^^103^ To address PML for the majority of the population currently harboring JCV, an antiviral is warranted.

We have demonstrated that the CRISPR/Cas9 system can interrupt JCV pathogenesis. This approach has the potential to remove actively replicating viruses—treating patients with PML—as well as persistent quiescent virus—in individuals at risk of developing PML due to anticipated immune compromise. We utilized multiple *in vitro* models (monolayer SVGA and three-dimensional COs) to determine the efficacy of a dual-gRNA CRISPR antiviral in reducing JC viral load but recognized the primary limitation of this study is translatability. Use of a three-dimensional, heterogeneous cell population does not account for the multisystem complexities of an animal model. The creation of an *in vivo* model for the investigation of JCV is an essential advancement in preclinical study. Due to site and species specificity of JCV, humanized mouse models may be the ideal future direction to further validate the safety and efficacy of the dual-gRNA CRISPR antiviral discussed here.^64^^,^^104^

## Materials and Methods

### Creation and packaging of CRISPR constructs

For the initial single-, dual-, and triple-gRNA constructs, gRNAs were first multiplexed into a pX601 vector using previously published techniques by Yin et al.^105^ The gRNA expression cassette was then sub-cloned into the pPapi vector through sticky-end ligation with T4 ligase (New England Biolabs) at the KpnI (New England Biolabs) and EcoRI (New England Biolabs) sites. The rearranged “split” construct was produced through sticky end ligation of the VP1 gRNA cassette into the XbaI (New England Biolabs) site of the single-gRNA construct already containing the LT-Ag gRNA at the KpnI/EcoRI site. Plasmids were confirmed by Sanger sequencing prior to cloning in High Efficiency Stable Competent *E. coli* (New England Biolabs). DNA was then extracted using Plasmid Plus Maxi Kit (QIAGEN) and reconfirmed by Sanger sequencing.

Packaging was performed using 293T/HEK cells and the *Lentiviral Production Workflow* for Lipofectamine 3000 Transfection Reagent (Invitrogen) with psPAX2 and pCMV-VSV-G as packaging and envelope helper plasmids, respectively. The ratio of construct DNA to psPAX2 was 3:1, whereas the ratio of construct DNA to pCMV-VSV-G was 3:2 by weight. Lentivirus-containing medium was then concentrated by ultracentrifugation per the protocol described by Kutner et al.^106^ Infectious units by volume were quantified by plaque-forming units as described in the aforementioned *Lentiviral Production Workflow* with 72-h transduction in polybrene-containing media (8 μg/mL) and 5-day puromycin titer (0.1 μg/mL) prior to crystal violet staining for manual counting (0.5% crystal violet in 25% methanol).

pX601 was a gift from Feng Zhang (Addgene plasmid # 61591; http://n2t.net/addgene:61591; RRID:Addgene_61591).^98^ The plasmid pPapi was a gift from John Doench & David Root (Addgene plasmid # 96921; http://n2t.net/addgene:96921; RRID:Addgene_96921).^60^ psPAX2 was a gift from Didier Trono (Addgene plasmid #12260; http://n2t.net/addgene:12260; RRID:Addgene_12260); pCMV-VSV-G was a gift from Bob Weinberg (Addgene plasmid #8454; http://n2t.net/addgene:8454; RRID:Addgene_8454).^107^

### Cell culture management and differentiation

All cell culture and accompanying analysis was performed in appropriate sterile conditions per government and institutional standards. Regular mycoplasma testing (Lonza) of culture media was performed at time of removal from storage as well as at initial seeding of experiments. All cell culture incubations were performed at 37°C and 5% CO_2_ in the presence of an autoclaved water pan for consistent humidity.

Two human cell lines transformed by SV40 large tumor antigen were originally purchased from ATCC. SVGA cells (ATCC CRL-8621) are fetal astroglia used for infection studies and the creation of novel construct-expressing cell lines. 293T/HEK cells are epithelial-like kidney cells and are used for the production of lentiviral vectors. Both were maintained in Dulbecco’s Modified Eagle Medium (Gibco) enriched with 10% Heat Inactivated Fetal Bovine Serum (Gibco) and 0.1% Penicillin/Amphotericin B (Gibco). Antibiotic/antimycotic mixture was removed during experiments.

Induced pluripotent stem cells were obtained from the Comprehensive NeuroHIV Center at Lewis Katz School of Medicine and developed into cerebral organoids using the STEMdiff Cerebral Organoid Kit (STEMCELL Technologies). In addition to incubation conditions described above, organoids matured on an orbital shaker at 75 rpm. Following 40 days of development, additional maturation was performed using STEMdiff Cerebral Organoid Maturation Kit (STEMCELL Technologies).

Cell counting for seeding and MOI calculations was performed using trypsinized single-cell solution stained with trypan blue (Gibco). Manual counting was performed on hemocytometer (Hausser Scientific) using light microscope at 10x magnification.

### Infection, transfection, and transduction

Handling of infectious materials, including Mad-1 viral stock, was performed in appropriate biosafety level 2+ conditions in accordance with all requisite governmental and institutional standards. Mad-1 virus, originating in pBluescript (KS) vector, was produced as described previously.^108^ To calculate multiplicity of infection, qPCR was used to quantify genomic copy number by volume. DNA was isolated from media using the Virus Mini Kit (NucleoSpin); concentration and purity were assessed by Nanodrop 2000 Spectrophotometer (Thermo Scientific). Primers and probe were designed for the 1,526–1,632 span of the Mad-1 genome (Table S2). Genomic copy number was determined from a standard curve generated by serial dilutions of the parent vector, pBluescript(KS).

Infections were performed in half levels of OptiMEM (Gibco)-reduced serum media for 6 h before an equal part of culture media (DMEM +10% FBS or CO media) was added for overnight incubation. After a total of 24 h of exposure to the virus, media was aspirated and replaced with fresh culture media.

Transfections were performed using Lipofectamine 3000 reagent as described in its accompanying manual. A minimum incubation period of 48 h from initiation of transduction was observed prior to harvest or use of transfected cells.

Transductions were performed in half levels of culture media enriched with 8 μg/mL polybrene (Sigma-Aldrich) for 6 h before an equal part of fresh media was added for overnight incubation. After a total of 24 h exposed to the virus, media was aspirated and replaced with fresh culture media. A minimum incubation period of 72 h from initiation of transduction was observed prior to harvest or selection of transduced cells or organoids.

### Cell line production, selection, and clonal expansion

SVGA cells were transduced as described above with lentivirus packaged pPapi (Cas9-only) or the LTAg+VP1 rearranged vector at MOI = 1. Following 72 h of transduction, media was replaced with DMEM containing 10% FBS and 1 μg/mL puromycin (Sigma-Aldrich). Cells were maintained in puromycin-containing media for 10 days prior to expansion in normal culture media. Expression of SaCas9 mRNA, LT gRNA, and VP1 gRNA was confirmed using RT-qPCR as described below. Protein expression was also confirmed by western blot as described below.

### Sample extraction from media and cell lysate

DNA and RNA were extracted from cell media using the Virus Mini Kit (NucleoSpin) at a 1:1 ratio by volume. Cells in monolayer were harvested through trypsinization (Gibco), whereas organoids were washed with PBS before being transferred to appropriate lysate buffer. DNA extraction from cells was performed using QIAmp DNA Miniprep Kit (QIAGEN). RNA extraction from cells was performed using Monarch Total RNA Miniprep Kit (New England Biolabs). Nucleic acid concentrations and purity were quantified using Nanodrop 2000 Spectrophotometer (Thermo Scientific). Protein extraction was performed using Pierce RIPA Lysis and Extraction Buffer (Thermo Scientific) in combination with 1% Protease Inhibitor Cocktail (Sigma-Aldrich). Protein concentration was quantified using Bradford Reagent (bioWORLD) and Multiskan FC Microplate Photometer (Thermo Scientific).

### Quantification and observation of DNA

DNA was quantified by qPCR using Luna Universal Probe qPCR Master Mix (New England Biolabs). Novel primers and accompanying probes were designed and employed (Table S2). A standard curve was generated through serial dilutions of a known concentration of pBluescript(KS) plasmid as described above.

Excision PCR was performed using novel primers designed upstream of LT-Ag and VP1 gRNA sites with Terra PCR Direct Polymerase (Takara) and 10 ng of DNA (Table S2). Electrophoresis was performed using a 1% agarose cast with 0.005% ethidium bromide. Gel electrophoresis was run in pre-chilled TAE buffer at a constant voLT-Age of 115V for 30 min before immediate analysis under ultraviolet light. Images were obtained using GelDoc-It2 Imager (Analytik Jena) and accompanying software.

Bands from gel electrophoresis were identified by UV for gross isolation using sterile, single-use razorblade. PCR products were then purified by NucleoSpin Gel and PCR Clean-Up kit. Sanger sequencing was ordered through Genewiz from Azenta Life Sciences using primers described above. Copy number variation was acquired through Synthego Performance Analysis, ICE Analysis. 2019. v3.0. Synthego; [Dec 2024].

### Quantification of RNA

For gRNA expression, extracted RNA was quantified using a previously validated reverse primer and probe for the gRNA scaffold with gRNA-specific forward primers (Table S2). For Cas9 mRNA, the aforementioned primers and probe for pPapi SaCas9 were used. Quantification was performed using Luna Universal One-Step RT-qPCR Kit (New England Biolabs) and analyzed using the LightCycler 96 System (Roche). Primers and probe for β-actin were used as a reference intrinsic to each reaction (Table S2).

### Quantification and observation of proteins from lysate

Western blotting was performed using hand cast 10% polyacrylamide gels and mini-PROTEAN Tetra cell (Bio-Rad). A 1.5 mm comb (Bio-Rad) containing 10 or 15 wells was molded in 5% stacking gel for loading. Protein samples were loaded at 20 μg per well with water dilution to a standard volume. Samples were combined with Laemelli Dye (2X) before incubation at 95°C for 15 min. A 50:50 water:dye combination was used to fill empty wells to ensure even distribution during run. Gels were run in 1X SDS buffer at constant voltage of 115 V for 1 h. Gels were transferred to 0.45 nM nitrocellulose membranes (Thermo Scientific) at constant current of 50 mA overnight (18 h) at 4°C using Mini Trans-Blot cell with PowerPac HC Power Supply (Bio-Rad).

Blocking was performed in 5% milk/PBS-T for 30 min. Primary antibodies were diluted at 1:1,000 in 2.5% milk/PBS-T for 2 h unless otherwise specified. Mouse antibodies include SaCas9 (GenScript), SV40 LT-Ag (Millipore), VP1 (Safak Lab, 1:250, 1% milk/PBS-T, overnight at 4°C), and loading control αTubulin (Invitrogen). Rabbit antibodies include agnoprotein (Excision). Membranes were washed three times for 5 min in PBS-T before secondary antibody exposure was performed for 1 h at a dilution of 1:5,000 in 2.5% milk/PBS-T. Secondary antibodies (Alexa Fluor) included goat anti-mouse 680 and goat anti-rabbit 800. Membranes were, again, washed three times for 5 min in PBS-T before imaging and analysis using Odyssey CLx imaging system (LI-COR Biotech) and accompanying software.

### Immunofluorescent imaging and quantification of proteins from whole organoids

Cerebral organoids were processed as outlined in the STEMCELL technical bulletin *Cryogenic tissue processing and section immunofluorescence of cerebral organoids*. Optimal cutting temperature compound (Tissue-Tek) was used in place of gelatin for snap freezing. Cryosectioning was performed using Microm HM505E with −20°C box temperature and 10 μm section thickness. Primary mouse antibodies and their dilutions include Olig2 (1:250, Takara), Map2 (1:500, Takara), GFAP (1:500, Takara), LT-Ag (1:100, Millipore), and VP1 (1:50, Abcam). Primary rabbit antibodies and their dilutions include agnoprotein (1:100, Excision). Secondary antibodies (Alexa Fluor) were all diluted 1:500 and included donkey anti-mouse 488, donkey anti-mouse 555, and donkey anti-rabbit 488. Mounting was performed in DAPI-containing VECTASHIELD HardSet media (Vector), set overnight at 4°C, and then immediately imaged using All-in-One Fluorescence Microscope (KEYENCE) and accompanying software.

### Statistical analysis

Student’s t tests and ANOVA were performed with accompanying post hoc analysis. Statistical significance was considered for *p* values <0.05, and degree of significance was ranked: ∗∗∗∗*p* < 0.0001, ∗∗∗*p* < 0.001, ∗∗*p* < 0.01, and ∗*p* < 0.05. Graphical data plot the mean with bidirectional bars representing standard error of the mean. Analysis and display were achieved using GraphPad Prism (v10.4.1).

## Data availability

All data in this manuscript are available from the authors on reasonable request.

## Acknowledgments

Our sincere thanks to past and present members of the Department of Microbiology, Immunology and Inflammation and the Center for Neurovirology and Gene Editing for support and insight. Statistical analysis and graph generation were performed using Excel and GraphPad Prism software. Viral genome and CRISPR construct sequence mapping was displayed using SnapGene software. Bioinformatic analyses were conducted with *Benchling* software utilizing viral and human genomes provided via NIH BLAST programs. A.R. was supported by the 10.13039/100000025National Institute of Mental Health of the 10.13039/100000002National Institutes of Health under Award Number T32MH079785. The content is solely the responsibility of the authors and does not necessarily represent the official views of the National Institutes of Health. Prior to issuing a press release concerning the outcome of this research, please notify the NIH awarding IC in advance to allow for coordination.

## Author contributions

K.K., I.K.S., and A.R. conceived the idea and designed the experiments. A.R., S.L., C.C., and H.L. conducted experiments. S.C., A.B., H.S.W., and A.R. produced model materials (iPSCs, COs, Mad-1). A.R., I.K.S., and K.K. interpreted data and wrote the manuscript.

## Declaration of interests

A.R., S.L., C.C., H.L., S.C., A.B., I.K.S., and H.S.W. declare no conflict of interest. K.K. is named inventor on patents that cover the viral gene editing technology that is the subject of this article. K.K. is a co-founder, board member, scientific advisor, and holds equity in Excision BioTherapeutics, a biotech startup that has licensed the viral gene editing technology from Temple University for commercial development and clinical trials.

## Supplemental information

Document S1. Figures S1–S4 and Tables S1 and S2

Document S2. Article plus supplemental information
