# Supplementary material for: CRISPR antiviral inhibits neurotrophic JC polyomavirus in 2D and 3D culture models through dual-gRNA excision by SaCas9
Source: Mol Ther Nucleic Acids. 2025 May 14;36(2):102556. doi: 10.1016/j.omtn.2025.102556 (PMC12159223; doi:10.1016/j.omtn.2025.102556)
Supplement: Document S1. Figures S1–S4 and Tables S1 and S2 [file mmc1.pdf]

OMTN, Volume *36*

Supplemental information

CRISPR antiviral inhibits neurotrophic JC polyomavirus in 2D and 3D culture models through dual-gRNA excision by SaCas9

Angela Rocchi, Shuren Liao, Hong Liu, Chen Chen, Senem Çakır, Anna Bellizzi, Hassen S. Wollebo, Ilker K. Sariyer, and Kamel Khalili

Table S1: Sequence, sensitivity, and specificity of gRNAs designed with *Benchling*

Target	Sequence (5'-3')	PAM	On-Target	Off-Target
NCCR	TGTATATATAAAAAAAAGGGA	AGGGAT	53.1	72.0
LTAg	GTCATGCTCCTTAAGGCCCCC	CTGAAT	48.7	91.0
VP1	GGGTTGACTCAATTACAGAGG	TAGAAT	50.7	88.2

Table S2: Primer and probe sequences used for PCR and sequencing

Target	Role	Sequence (5'-3')	Function
JCV	Top Primer	CTTATAAGAGGAGGAGTAGAAGTT	qPCR<br>Viral genomic copy number
	Bottom Primer	AGATGCTCATCTGGGTCA	
	Probe (FAM)	AGCATTCTACCTCTGTAATTGAGTCAACCC	
SaCas9<br>(pPapi)	Top Primer	TTGTCCAACTTTTTCCACTCC	RT-qPCR<br>Treatment expression
	Bottom Primer	GACTTCATCAACCGCAACC	
	Probe (FAM)	CGTCCTCCGCATGGTGCTTGTAGCCCTT	
LTAg gRNA	Top Primer	CACCGTCATGCTCCTTAAGGC	
VP1 gRNA	Top Primer	CACCGGGTTGACTCAATTACAG	
gRNA Scaffold	Bottom Primer	CGCCAACAAGTTGACGAGATAA	
	Probe (FAM)	CAGAATCTACTAAAACAAGGCAAA	
Excision	LTAg Primer	AGATCTGCATGCATTCCTCAGT	ePCR<br>Dual-gRNA excision
	VP1 Primer	CCCAAATGTGCAATCTGGTGAA	
Sequencing	LTAg Primer	TCCTGAAAGTCTAAGTACATGCCC	Sanger sequencing<br>Alignment and ICE analysis
	VP1 Primer	GGAATTCTGGCCACACTGTAAC	
βActin	Top Strand	GCATCCTCACCCTGAAGTA	PCR and RT-qPCR<br>Intrinsic control
	Bottom Primer	CACGCAGCTCATTGTAGAAG	
	Probe (HEX)	ACCAACTGGGACGACATGGAGAAA	

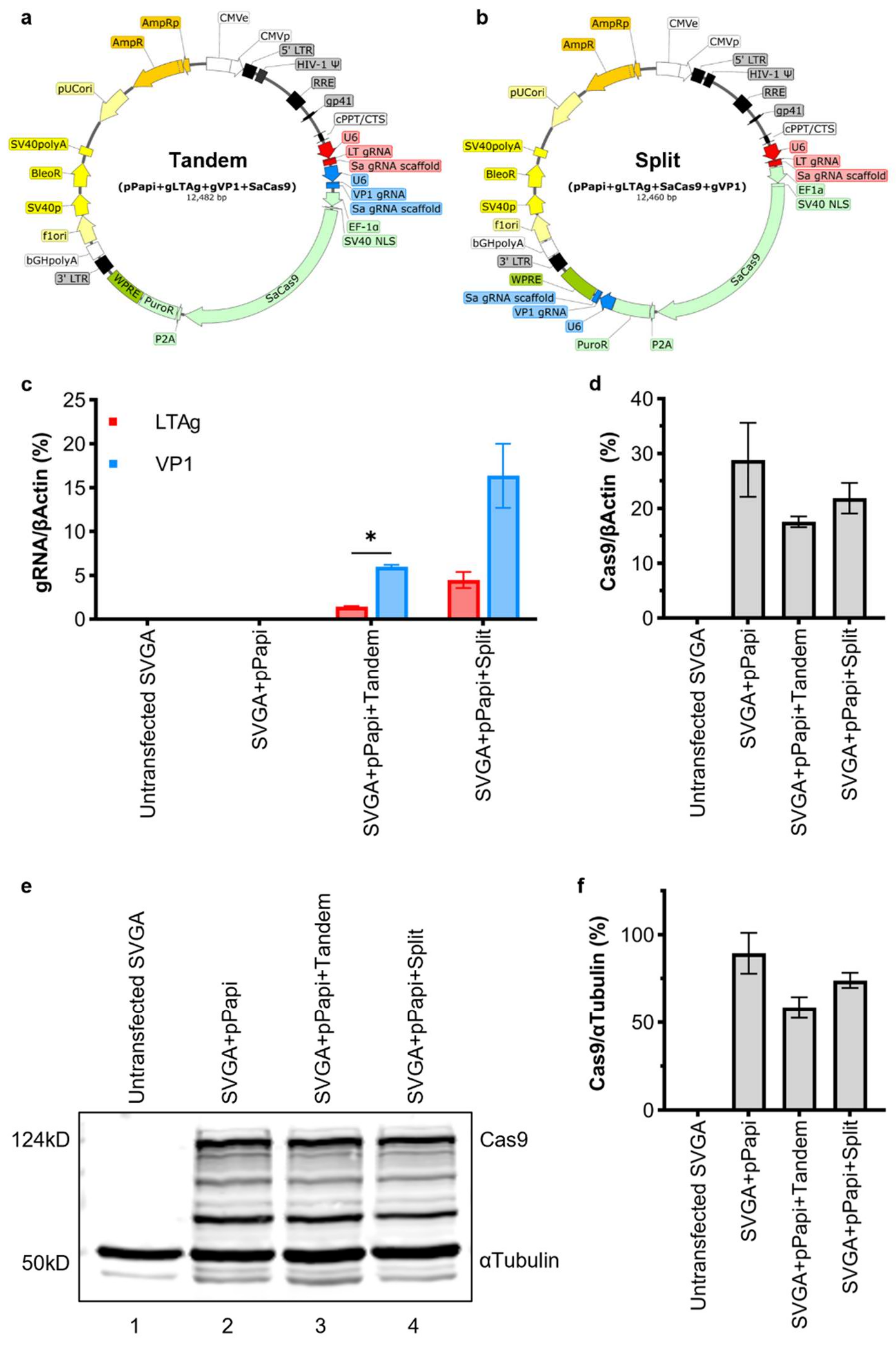

Figure S1: Dual gRNA expression is improved when U6 expression cassettes are separated by a EF1α expression cassette in pPapi vector plasmid

a. Maps of CRISPR plasmids encoding SaCas9 and puromycin resistance gene under shared EF1α promoter (green) with gRNAs targeting LTAg (red) and VP1 (blue) under separate U6 promoters. Lentiviral packaging components (black) and bacterial selection components for cloning (yellow). Cassettes for gRNA expression ordered in “Tandem” preceding SaCas9 cassette.
b. Map of CRISPR plasmid described above unchanged from shared parental pPapi plasmid with the exception of the gRNA cassettes, which are “Split” by the SaCas9 cassette.
c. RT-qPCR quantifying level of gRNAs targeting LTAg (red) and VP1 (blue) in untransfected SVGA cells (SVGA), parental plasmid Cas9-only transfected SVGA cells (SVGA+pPapi), or construct transfected SVGA cells with gRNA in tandem (SVGA+pPapi+Tandem) or split (SVGA+pPapi+Split) organization. Both LTAg and VP1 gRNAs increased

threefold in Split construct compared to Tandem. Statistical significance observed between LTAg and VP1 gRNAs for Tandem construct (* = $p = 0.0023$, $n = 8$) but not Split construct (ns = $p = 0.0875$, $n = 8$).

d. RT-qPCR measurement of SaCas9 RNA expression relative to βactin standard. No significant difference observed across transfection conditions described above.

e. WB displaying Cas9 protein expression relative to αTubulin loading control following transfection with plasmids described above.

f. Quantification of WB described above indicates no significant difference in Cas9 protein levels across transfection conditions described above.

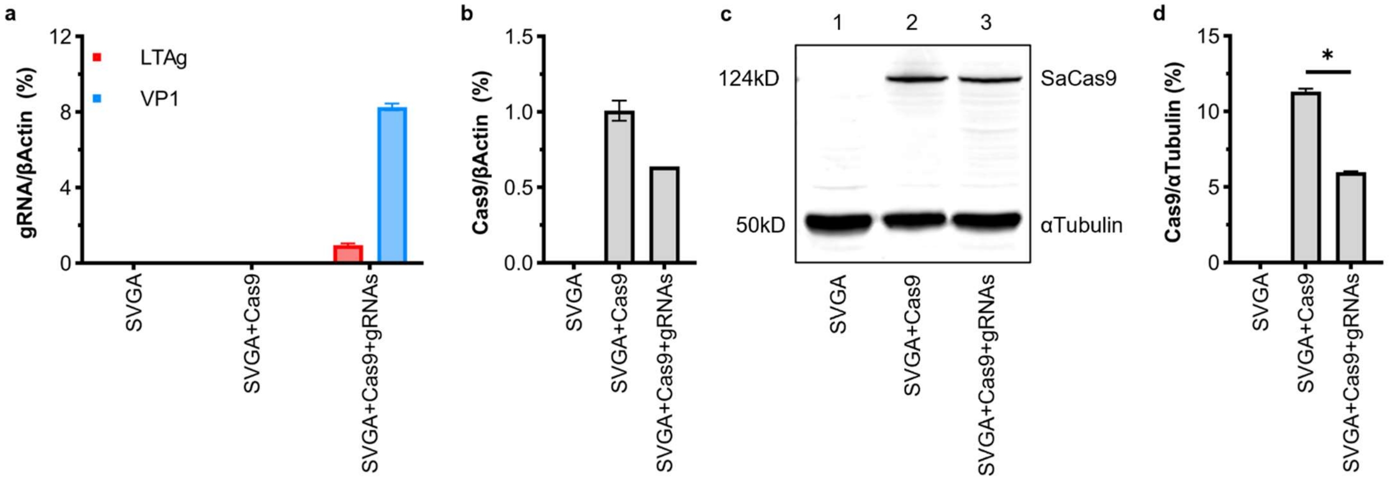

Figure S2: Construct expression in puromycin-selected cell lines

a. RT-qPCR confirming expression of gRNAs targeting LTAg (red) and VP1 (blue) in parental SVGA cell line (SVGA), stable cell line produced by selection for Cas9-only construct expression (SVGA+Cas9), or selection for the full treatment construct expressing LTAg and VP1 gRNAs in split organization (SVGA+Cas9+gRNAs).

b. RT-qPCR confirming expression of SaCas9 RNA for cell lines described above.

c. WB displaying Cas9 protein expression relative to αTubulin loading control of cell lines described above.

d. Quantification of WB described above reveals a significant decrease in Cas9 protein levels of full-construct expressing cells compared to Cas9-only cells (* = p = 0.0405).

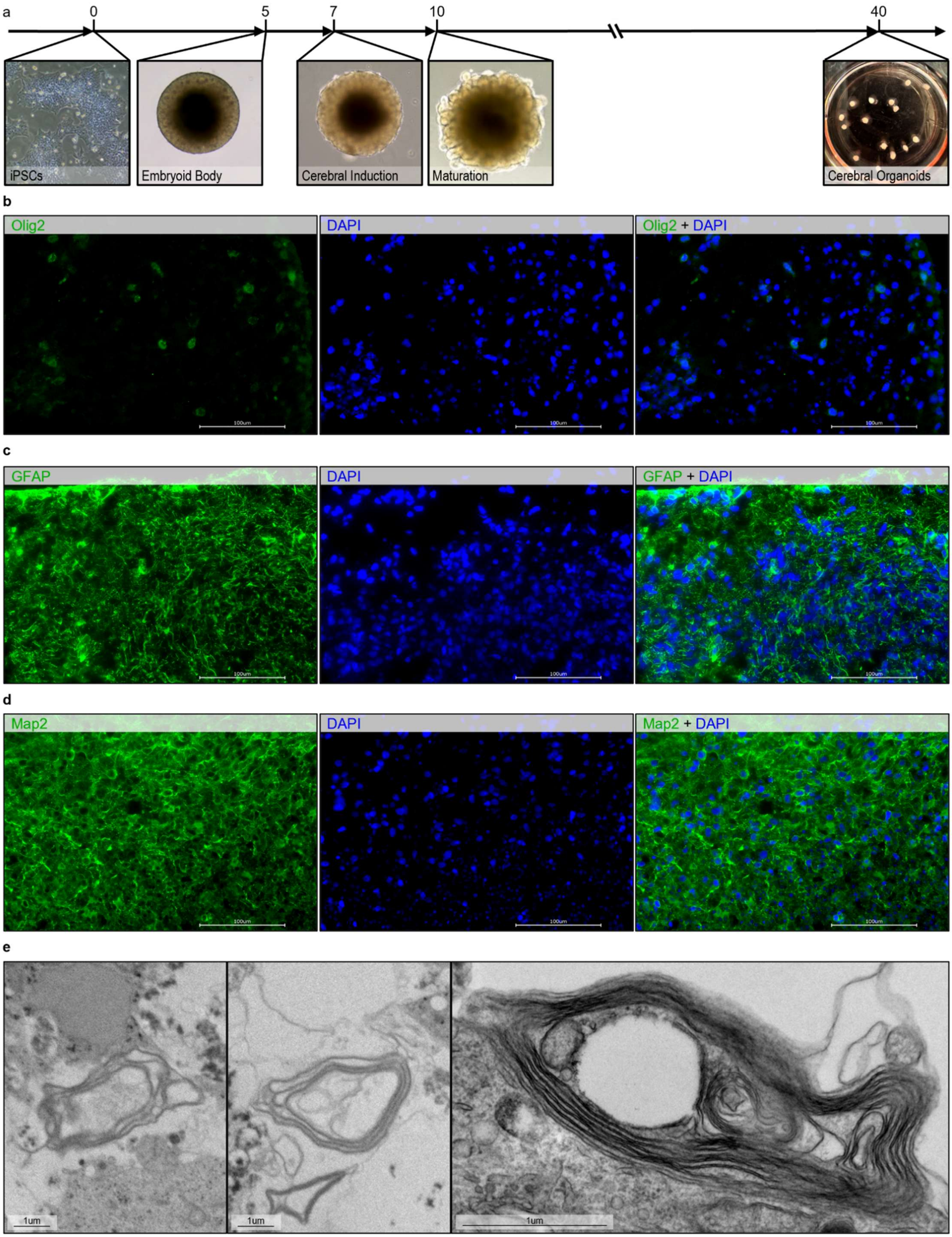
a
0
5
7
10
40
iPSCs
Embryoid Body
Cerebral Induction
Maturation
Cerebral Organoids
b
Olig2
DAPI
Olig2 + DAPI
c
GFAP
DAPI
GFAP + DAPI
d
Map2
DAPI
Map2 + DAPI
e
1um
1um
1um

Figure S3: Development and characterization of cerebral organoids using multiple imaging modalities

a. Developmental timeline for the creation of three-dimensional cerebral organoids with major morphological milestones demarcated with 10x light microscopy images.
b. Immunofluorescent labeling of organoids 120 days post differentiation from iPSCs depicting heterogeneous cell types of matured cerebral organoids including oligodendrocyte cell bodies (Olig2) with nuclear control stain (DAPI).
c. Immunofluorescent labeling of astrocyte processes (GFAP) with nuclear control stain (DAPI) from COs described above.
d. Immunofluorescent labeling of neuron dendrites (Map2) with nuclear control stain (DAPI) from COs described above.
e. Electron microscopy of COs in conditions described above showing layering consistent with myelin sheaths with varying degrees of compacted sheathing.

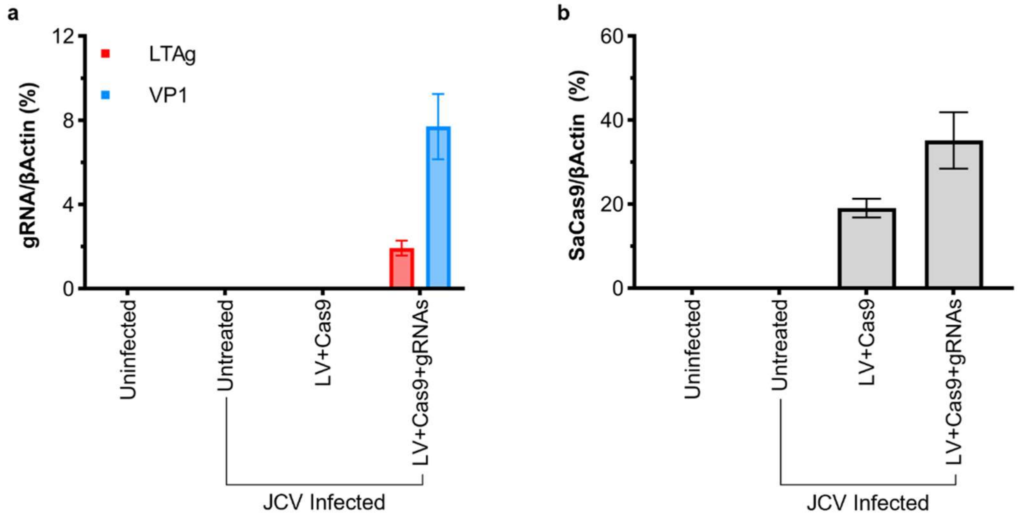

Figure S4: Confirmation of construct expression in COs 14 days post-transduction with CRISPR lentivirus targeting JCV LTAg and VP1

a. Quantification of gRNA expression levels by RT-qPCR for LTAg (red) and VP1 (blue) gRNAs in CO that are uninfected and untransduced (Uninfected); infected with Mad-1 but untransduced by lentivirus (Untreated); infected with Mad-1 and transduced by Cas9-only lentivirus (LV+Cas9); or infected with Mad-1 and transduced with CRISPR lentivirus targeting LTAg and VP1 (LV+Cas9+gRNAs). No significant difference was identified between LTAg and VP1 gRNAs of the treatment group (ns = $p = 0.3910$, $n = 12$).

b. RT-qPCR quantifying SaCas9 RNA for the conditions described above was not significantly different between Cas9-only and full CRISPR transduced organoids (ns = $p > 0.9999$, $n = 24$).
